# Enzyme-Assisted Extraction and Preparation of Saponin Microcapsules and Gelatin Gummies: Characterization and In Vitro Digestion

**DOI:** 10.3390/foods15081332

**Published:** 2026-04-11

**Authors:** Yehui Zhou, Jie Long, Enduo Ma, Xia Zheng, Xingfei Li, Zhengyu Jin

**Affiliations:** 1 The State Key Laboratory of Food Science and Resources, Jiangnan University, 1800 Lihu Road, Wuxi 214122, China; 6230111158@stu.jiangnan.edu.cn (Y.Z.); jielong@jiangnan.edu.cn (J.L.); lixfei2019@jiangnan.edu.cn (X.L.); 2 School of Food Science and Technology, Jiangnan University, 1800 Lihu Road, Wuxi 214122, China; amos168@amos-sweets.com; 3 BioBor (Guangdong) Health Technology Co., Ltd., 180 Shengli South Road, Jiangmen 529000, China; xia.zheng@amos-sweets.com

**Keywords:** dual-enzyme extraction, saponin, microcapsule, controlled release

## Abstract

Saponins, the primary bioactive constituents with immunomodulatory activities in Baoyuan decoction—a traditional Chinese medicine formula composed of ginseng, astragalus, licorice, and cinnamon—are limited by low extraction yield, poor stability, and easy degradation. In this study, cellulase and pectinase were used for the extraction of saponins from Baoyuan decoction and optimized by response surface methodology. Subsequently, the optimal extracts were microencapsulated by spray drying with soy protein isolate (SPI) or high-oleic acid soy protein isolate (HOSPI) and pectin (PE) as composite wall materials, followed by application evaluation in gummies and in vitro digestion. After optimization, the total saponin yield was 63.68 ± 0.15 mg/g. HOSPI-PE microcapsules (HBP) had a higher encapsulation efficiency (90.38%), smaller particle size, and lower hygroscopicity than SPI-PE ones (SBP). Furthermore, both microcapsules showed good stability during storage and controlled release, with 60.9% of saponins in SBP and 65.8% in HBP being delivered to the intestinal phase during in vitro digestion of microparticles. When applied in gummies, microcapsule gummies retained satisfactory sustained-release in vitro digestion (23.0% released in the stomach and 66.2% in the small intestine). In contrast, the unencapsulated gummies exhibited a burst release (74.4%) at 30 min in gastric digestion. This study provides theoretical and technical insights into the development of plant-derived functional foods and promotes the practical application of microencapsulation in functional gummy candies.

## 1. Introduction

Nowadays, the “sub—health” state has become a prevalent public health issue, characterized by fatigue, lassitude, and low immunity [1]. Baoyuan decoction, a classic prescription in traditional Chinese medicine, is composed of four medicine–food homologous components, including ginseng, astragalus, licorice, and cinnamon. It exerts the effects of improving overall physiological resilience and regulating immunity in the human body [2]. Among the components, ginseng serves as the core of immune regulation, and its bioactivity mainly relies on steroidal compounds such as ginsenosides [3], which possess multiple bioactivities including immune regulation, antifatigue, and antioxidation [4]. Yet, traditional approaches for plant extraction are plagued by low extraction efficiency. Moreover, bioactive components like saponins and polysaccharides are sensitive to light, heat, and oxygen, which makes them prone to degradation during processing and storage [5]. More critically, although the digestive behavior of saponins in the stomach is structure-dependent and cannot be generalized, some kinds of saponins are still susceptible to degradation under gastric acid conditions, limiting their practical application [6,7]. Therefore, developing safe and efficient plant extraction methods and improving the stability and bioaccessibility of plant-derived bioactive components are of significant practical value.

Traditional plant extraction approaches have drawbacks such as long extraction cycles, high solvent consumption, low extraction efficiency, cumbersome technological processes, and high extraction costs [8]. The enzymatic extraction method operates on the core principle of selecting suitable bioenzymes according to the plant species, medicinal parts, and characteristics of target components. Through mild enzymatic hydrolysis, it disrupts and degrades the plant cell wall structure, thereby promoting the liberation and release of bioactive components [9,10]. This method boasts advantages of safety, high efficiency, simple and controllable processes, and exhibits excellent potential for industrialization [11].

Microencapsulation refers to the technology of wrapping active core materials with a layer of wall materials to form micron-sized particles. This physical isolation can protect active ingredients from oxygen, light, and other external interferences, reduce oxidation and degradation, mask off-flavors, and improve the stability and application performance of the active substances [12]. In addition, this process can improve the water solubility and dispersibility of active ingredients, mask undesirable flavors, achieve the targeted controlled release of active components, and ultimately boost bioaccessibility [13]. Several microencapsulation techniques have been developed, including spray drying, freeze drying, complex coacervation, ionic gelation, and liposome encapsulation [14]. Among these methods, spray drying is particularly suitable for the encapsulation of plant-derived extracts, owing to its high efficiency, low cost, and ease of industrialization. In the process of spray-drying microencapsulation, the selection of appropriate wall materials is a critical step, since wall materials exert a significant effect on the yield, morphology, stability, and encapsulation efficiency of the final products [15].

Proteins and polysaccharides are frequently employed as composite wall materials, which exhibit distinct advantages. As carriers for nutrients, protein particles possess favorable biocompatibility. In addition, proteins are amphipathic and can serve as encapsulation matrices for both hydrophobic and hydrophilic functional ingredients [16]. Soy protein isolate (SPI) is inexpensive and readily available, with excellent emulsifying, gelling and film-forming properties, making it an ideal candidate for the construction of delivery carriers. In addition, high-oleic acid soy protein isolate (HOSPI) exhibits enhanced solubility and stability, which can further improve its film-forming performance [17]. Pectin, a plant-derived polysaccharide, is resistant to gastric digestive enzymes; upon entering the intestinal tract, pectin molecules can be degraded by intestinal microorganisms [18]. This digestive property can prevent active ingredients from being destroyed by gastric acid in the stomach. Furthermore, it also enables the effective entrapment of active ingredients to isolate them from harmful external conditions including oxygen, light, and temperature [19].

Although Baoyuan decoction is rich in bioactive functional components, its application in functional foods is limited by low extraction efficiency, the instability of active compounds, and poor palatability of the extract. To solve these problems, this study employed cellulase and pectinase to extract the active components, utilized spray-drying microencapsulation technology to enhance the stability of active components, and further processed them into functional gummies with good palatability. The purpose is to provide support for the innovation and development of medicinal herb-derived functional foods with high stability and good palatability.

## 2. Materials and Methods

### 2.1. Materials

Ginseng (*Panax ginseng* C. A. Mey.), astragalus (*Astragalus membranaceus* var. *mongholicus* (Bge.) Hsiao), licorice (*Glycyrrhiza uralensis* Fisch.), and cinnamon (*Cinnamomum cassia* (L.) J. Presl) were purchased from Beijing Tong Ren Tang Technology Development Co., Ltd., Wuxi, China. Batch numbers: ginseng (250301), astragalus (250301), licorice (250214), and cinnamon (250214). Cellulase (50 U/mg), pectinase (50 U/mg), soy protein isolate, ginsenosides Re, Rg1, and Rb1 (HPLC ≥ 98%), and pectin were all supplied from Shanghai Yuanye Bio-Technology Co., Ltd., Shanghai, China. High-oleic acid soy protein isolate was supplied by Shandong Yuwang Industrial Co., Ltd., Dezhou, China; high-oleic acid soybean protein isolate is extracted from high-oleic acid soybean plants, with more α-helix and random coil compared to soybean protein isolate.

### 2.2. Preparation of Plant Enzymatic Hydrolysate

Ginseng, astragalus, licorice, and cinnamon were first ground into powders and then sieved through a 60-mesh sieve. The powder (ginseng: astragalus: licorice: cinnamon = 3.73:7.46:1.87:0.75, g) and deionized water were combined at a solid–liquid ratio of 1:20 (*w*/*v*). Subsequently, 2% of cellulase (*w*/*w*) and 2% of pectinase (*w*/*w*) were added. The mixture was magnetically stirred continuously in a 50 °C water bath for a duration of 2 h, followed by temperature elevation to 100 °C for further extraction for 40 min [20]. After centrifugation at 4000 rpm for 15 min, the supernatant was collected for subsequent experiments. A systematic investigation was conducted to assess the effects of several key factors on total saponin yield, encompassing cellulase amount, pectinase amount, solid-to-liquid ratio, temperature, pH, and enzymatic hydrolysis time. The conditions of the single-factor experiments are shown in Table 1. All enzymatic hydrolysis experiments were performed using an SHJ A6 magnetic stirring water bath (Changzhou Yineng Experimental Instrument Factory, Changzhou, China). Using ginsenoside Re as the standard, the total saponin content was determined by the vanillin-perchloric acid method [21]. The total saponin yield was calculated according to the following formula:

(1) Y (mg/g) = (C × V)/M_0_

where Y is the total saponin yield (mg/g); C is the measured saponin concentration in the sample solution (mg/mL); V is the volume of the extract solution (mL); and M_0_ is the mass of the powder (g).

### 2.3. Response Surface Methodology (RSM)

With the single-factor experiments having identified the optimal ranges of variables, a four-factor Box–Behnken Design (BBD) was implemented for in-depth analysis. Design-Expert 13.0 was used for experimental design and RSM data analysis, while Origin 2023 was employed for figure plotting. The four independent variables included enzymatic hydrolysis temperature (A), time (B), pH (C), and solid–liquid ratio (D) (Table 2).

### 2.4. Component Identification

Quantitative analysis of the main ginsenosides in the enzymatically hydrolyzed Baoyuan decoction was performed by HPLC, with slight modifications according to the Chinese Pharmacopoeia [22].

Preparation of sample solution was as follows: About 1.0 g of the freeze-dried powder of the reference substance of Baoyuan decoction was accurately weighed, then 50 mL of water-saturated 1-butanol was precisely added, and the mixture was allowed to stand overnight. It was subjected to ultrasonic treatment (power: 500 W; frequency: 40 kHz) for 30 min, then cooled and filtered. A 25 mL aliquot of the subsequent filtrate was transferred to an evaporating dish and evaporated to dryness. The residue was then dissolved in methanol and diluted to volume in a 5 mL volumetric flask to obtain the test solution.

Preparation of reference solution was as follows: ginsenoside Rg1, ginsenoside Re, and ginsenoside Rb1 were accurately weighed and dissolved in methanol to prepare a mixed solution containing 0.2 mg/mL. The solution was then shaken well to obtain the final solution.

The analysis was performed on a LC-20A system (Shimadzu, Kyoto, Japan) equipped with an ZORBAX SB-C18 column (Agilent, Santa Clara, CA, USA). The column temperature was maintained at 25 °C, with a flow rate of 1.0 mL/min and an injection volume of 10 μL; detection wavelength was set at 203 nm. Gradient elution was performed according to Table 3.

### 2.5. Preparation of Plant Extract Microcapsules

The plant extract prepared under the aforementioned optimal conditions was mixed with 0.8% (*w*/*v*) soy protein isolate, followed by magnetic stirring (SHJ-A6, Changzhou Yineng Experimental Instrument Factory, Changzhou, China) at 600 rpm for 20 min at room temperature. Subsequently, 0.2% (*w*/*v*) pectin was added, and continuous stirring was maintained for 1 h. The mixture was then hydrated overnight at 4 °C [16]. Microencapsulation was performed using a spray dryer (MOBILE MINOR, GEA Engineering Technologies (China) Co., Ltd., Shanghai, China) under the following conditions: 180 °C inlet air temperature, 80 °C outlet air temperature, 3 m^3^/h atomization rate, and 20 mL/min feed rate [23]. Finally, the spray-dried microcapsule powder was placed in sealed bags and stored in airtight desiccators at room temperature.

### 2.6. Preparation of Microcapsule-Loaded Gummy

**Figure 1:**
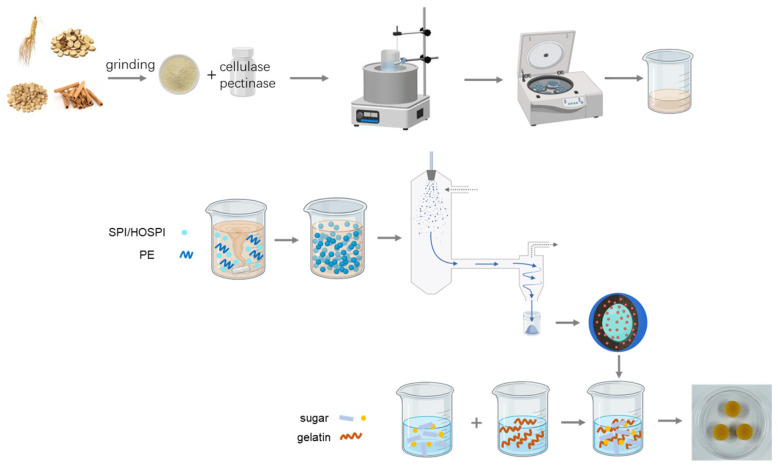
Schematic diagram of gummy candy preparation process. SPI: soy protein isolate; HOSPI: high-oleic acid soy protein isolate; PE: pectin.

Syrup was prepared via the mixing of malt syrup, sugar, and water, followed by heating to 118 °C. Gelatin underwent 30 min of hydration in cold water and 30 min of melting in a 60 °C water bath. The melted gelatin was added to the 100 °C syrup mixture, with 3 min of stirring to ensure homogeneity. The high-oleic acid soy protein isolate–pectin microcapsules were dissolved in water, and when the temperature dropped to 50 °C, the microcapsule solution was added to the sugar–gelatin mixture, followed by stirring for 5 min. The mixture was adjusted to pH 3.0 via the addition of citric acid, after which solution was poured into molds, cooled for 24 h, and then demolded [24]. Three types of gummies were prepared: blank gummy candies, gummy candies with water decoction, and HBP microcapsule-loaded gummy candies. A schematic diagram of gummy candy preparation process is provided in Figure 1.

### 2.7. Characterization

A Zetasizer Nano-ZS90 instrument (Malvern Instruments Ltd., Malvern, UK) was used to quantify particle size, PDI, and ζ-potential [25].

The FTIR spectra of samples were recorded on a Nicolet Nexus 470 FTIR spectrometer (Thermo Electron Corporation, Waltham, MA, USA) in the wavenumber range of 500–4000 cm^−1^, a resolution of 4 cm^−1^, and 32 scans performed per sample [26].

The crystalline structure was defined using a D2 PHASER X-ray diffractometer (Bruker, Karlsruhe, Germany). The test was performed under the conditions of 40 kV voltage and 40 mA current, with a scanning range of 5° to 40° [27].

The thermal stability of the microcapsules was evaluated using a thermogravimetric analyzer (TGA2, Mettler Toledo, Greifensee, Switzerland). A total of 4 mg of microcapsule samples were weighed and placed in a crucible. The samples were heated from 30 °C to 600 °C at a heating rate of 20 °C/min, with a nitrogen flow rate of 20 mL/min maintained throughout the test [27].

The morphological characteristics of the microcapsules were determined via a SU8220 scanning electron microscope (Hitachi High-Technologies, Tokyo, Japan). Samples were fixed on conductive adhesive tape, followed by gold sputtering treatment and scanning at an accelerating voltage of 5.0 kV [28].

### 2.8. Water Activity, Moisture Content, Solubility, and Hygroscopicity

A digital water activity meter (HD-4, Yunke Biotechnology Co., Ltd., Kunming, China) was employed to measure the water activity, the microcapsules were dried in an oven at 105 °C, and the moisture content was calculated according to the following equation:

(2) $$\mathrm{Moisture}\;\mathrm{content}\;(\%)\;=\;\frac{M_{1}-M_{2}}{M}\times 100$$

*M* is the mass of the microcapsules, and *M*_1_ and *M*_2_ are the total mass of the microcapsules plus the container before and after drying, respectively.

Solubility was determined following the method described by Liu et al. [29]. Two grams of microcapsules were added into 20 mL of ultrapure water, followed by continuous stirring for 30 min. After centrifugation at 3000 rpm for 15 min, the insoluble solids were transferred into a crucible and dried to a constant weight at 105 °C. The solubility was calculated according to the following equation:

(3) $$\mathrm{Water}\;\mathrm{solubility}\;(\%)\;=\;(1-\frac{M_{2}-M_{1}}{\left(1-B\right)\times M})\times 100$$

where *M* is the mass of the microcapsules, *M*_1_ is the mass of the crucible, *M*_2_ is the mass of the crucible and the dried insoluble substances, and *B* is the moisture content of the sample.

The hygroscopicity was determined following the method described by Hu et al. [21], with minor modifications. After being weighed, the microcapsule samples were placed in a desiccator containing saturated sodium chloride solution (relative humidity: 75%) at 25 °C and reweighed after 7 days. The hygroscopicity was calculated according to the following equation:

(4) $$\mathrm{Hygroscopicity}\;(g/100\;g)\;=\;\frac{W_{2}-W_{1}}{W}\times 100$$

*W*, *W*_1_, and *W*_2_ are the mass of the microcapsules, the total mass of the microcapsules plus the Petri dish initially, and the total mass of the microcapsules plus the Petri dish after 7 days of incubation, respectively.

### 2.9. Encapsulation Efficiency (EE) of Microcapsules

Determination of total saponins on the microcapsule surface: An accurately weighed amount of 0.5 g microcapsules was rinsed repeatedly with methanol solution, followed by centrifugation at 4000 rpm for 10 min. Corresponding filtrate was gathered in a 10 mL volumetric flask. The absorbance was measured at a wavelength of 540 nm. The total saponin content of the microcapsule surface was calculated by applying the standard curve equation: 0.5 g of the enzymatically hydrolyzed extraction powder was accurately weighed and dissolved in methanol to a constant volume of 10 mL, and serial gradient dilutions were performed to plot the standard curve of total saponins in the methanol solution.

Determination of total saponins in the microcapsules: An accurately weighed amount of 0.5 g microcapsules was dissolved in deionized water and subjected to ultrasonic treatment for 30 min, then centrifuged at 4000 rpm for 10 min. The filtrate was gathered and moved into a 10 mL volumetric flask; subsequently, its absorbance was determined at a wavelength of 540 nm. The content of total saponins in the microcapsules was calculated using the standard curve equation [21]. The encapsulation efficiency of the microcapsules was calculated according to the following equation:

(5) $$\mathrm{EE}\;(\%)\;=\;\left(1-\frac{Total\;saponin\;content\;on\;the\;surface}{Total\;saponin\;content\;in\;the\;microcapsules}\right)\times 100$$

### 2.10. Storage Stability

The microcapsule powder was stored in a constant-temperature incubator at 50 °C in the dark for two weeks. Samples were collected on days 1, 3, 7, and 14 to evaluate the accelerated storage stability of the microcapsules [30]. The total saponin retention index was calculated according to the following equation:

(6) $$\mathrm{RI}\;(\%)\;=\;\frac{Total\;saponin\;content\;after\;treatment}{Initial\;total\;saponin\;content}\times 100$$

### 2.11. Gummy Texture

The texture of gummy candies was assayed using texture analyzer (TA.XT Plus, SMS, Godalming, UK) according to the analytical protocol established by Yang et al. [31].

### 2.12. In Vitro Digestion of Microcapsules and Gummies

The simulated in vitro digestion assays were performed for the oral, gastric and intestinal phases, respectively [32]. During simulated oral digestion, 1 g of the sample was dispersed in 10 mL of simulated oral fluid (containing 75 U/mL salivary amylase and 1.5 mg/mL mucin), followed by incubation at 37 °C and 150 rpm for 2 min. Then, 10 mL of the post-oral digestion solution was combined with 10 mL simulated gastric fluid (2000 U/mL pepsin and 60 U/mL gastric lipase). The mixture pH was adjusted to 3.0 with 2 M HCl, then incubated at 37 °C and 150 rpm for 2 h. After adjusting the gastric fluid pH to 7.0 with 2 M NaOH, 20 mL of simulated small intestinal fluid (containing 10 mM porcine bile and 100 U/mL porcine pancreatin trypsin) was added to the gastric digestion mixture. The pH of the mixed fluid was readjusted to 7.0, followed by incubation at 37 °C for 2 h with a stirring speed of 150 rpm.

### 2.13. Data Analysis

All experiments were performed in triplicate; results are presented as means ± standard deviations. The resulting data were analyzed using analysis of ANOVA, followed by Tukey’s test, and a value of *p* ≤ 0.05 was considered statistically significant.

## 3. Results and Discussion

### 3.1. Single-Factor Analysis

**Figure 2:**
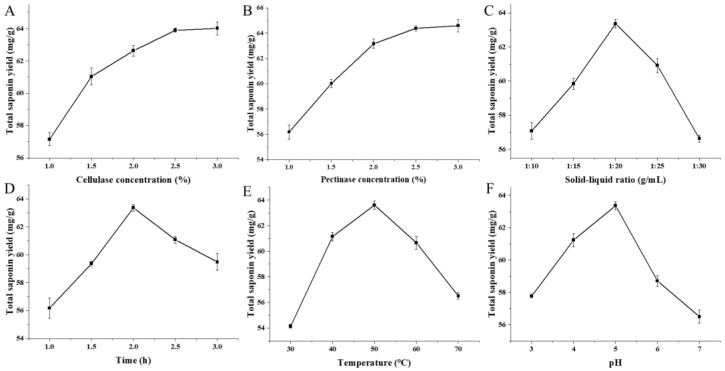
Effects of different extraction factors on total saponin yield. (**A**) Pectinase concentration; (**B**) cellulase concentration; (**C**) solid–liquid ratio; (**D**) time; (**E**): temperature; and (**F**) pH.

The effects of pectinase and cellulase concentration on the total saponin yield are shown in Figure 2A,B. With the increase in enzyme concentration, the total saponin yield increased rapidly and then tended to be stable, indicating that the enzyme concentration in the reaction system was saturated. Figure 2C illustrates the effect of different solid–liquid ratios on the total saponin yield. As the solid–liquid ratio increased, the total saponin yield increased and peaked at a ratio of 1:20. Further increases in the solid–liquid ratio led to a decrease in yield, as the relatively insufficient enzyme concentration compromised the hydrolysis reaction. The effect of different extraction times on the total saponin yield was investigated, and the results are presented in Figure 2D. The total saponin yield increased with the extension of the extraction time and reached the maximum at 2 h. Prolonging the extraction time further led to a decrease in yield, which might be attributed to the reduced enzyme activity caused by overlong enzymatic hydrolysis time or the degradation of saponin components under prolonged heating. In Figure 2E, the total saponin yield increased rapidly with the elevation of the enzymatic hydrolysis temperature and declined after peaking at 50 °C. This phenomenon is ascribed to the fact that cellulase and pectinase generally exhibit the highest activity at 50 °C, and their enzymatic activity decreases when the temperature exceeds the optimal range, which is consistent with the results reported by Liu et al. [33]. Figure 2F examines the effect of pH values on the total saponin yield. The yield increased with the rise in pH value and reached the maximum at pH 5. Within the pH range of 5–7, the total saponin yield decreased gradually, because the spatial structure of enzymes might change under different pH conditions, thereby altering their conformation and activity [34].

### 3.2. RSM Analysis

The Box–Behnken design (BBD) was used to optimize the enzymatic hydrolysis extraction process. With the total saponin yield defined as the response variable, the correlation between the predicted value and each influencing factor can be depicted by the following polynomial equation:

Y = 63.68 + 1.22A + 0.1383B − 0.6542C − 0.6058D + 0.3750AB − 0.2875AC − 0.0325AD + 0.8075BC − 0.0125BD + 0.7525CD − 2.48A^2^ − 2.61B^2^ − 3.36C^2^ − 4.45D^2^

The significance of the obtained experimental data was evaluated using analysis of variance (ANOVA), and this method was also utilized to examine the adequacy and fitness of the proposed model [35]. As can be seen from the results in Table 4, the high F-value (30.99) and low *p*-value (<0.0001) indicated that the regression model was highly significant. The non-significant lack of fit term (F-value = 12.66, *p*-value = 0.0754 > 0.05) demonstrated that the model was sufficient to predict the total saponin yield of the plant extract. With a high R^2^ value of 0.9731 and an adjusted R^2^ value of 0.9417, the model exhibited a robust correlation between the forecasted results and the actual experimental data. The low coefficient of variation (C.V. = 1.06%) and high adequate precision (Adeq Precision = 19.0434) reflected the extremely high accuracy and reliability of the experimental data. Overall, the fitted model could fully cover the scope of the experimental design and was proven to be an ideal model, which could be used to guide subsequent experiments.

**Figure 3:**
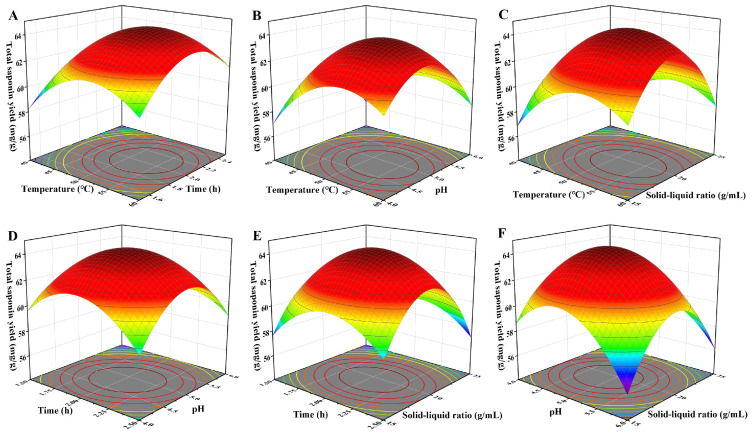
Three-dimensional response surface plots of total saponin yield. (**A**) 3D response surface plot of temperature and time (pH fixed at 5.0; solid–liquid ratio fixed at 1:20); (**B**) 3D response surface plot of temperature and pH (time fixed at 2 h; solid–liquid ratio fixed at 1:20); (**C**) 3D response surface plot of temperature and solid–liquid ratio (time fixed at 2 h; pH fixed at 5.0); (**D**) 3D response surface plot of time and pH (temperature fixed at 50 °C; solid–liquid ratio fixed at 1:20); (**E**) 3D response surface plot of time and solid–liquid ratio (temperature fixed at 50 °C; pH fixed at 5.0); and (**F**) 3D response surface plot of pH and solid–liquid ratio (temperature fixed at 50 °C; time fixed at 2 h).

The response surface plots more intuitively reflect the impacts of factors on the total saponin yield. A higher degree of steepness in the 3D response surface slope signifies a more notable influence of the independent variable on the yield [36]. In addition, whether the factors significantly influence the response value can be judged by the ellipticity of the corresponding contour lines [37]. In Figure 3, the effects of enzymatic hydrolysis temperature, pH, and the solid–liquid ratio on the total saponin yield are more significant than that of enzymatic hydrolysis time. Furthermore, the interactions between hydrolysis time and pH, as well as between pH and the solid–liquid ratio, are significant. The analysis results of RSM are highly consistent with the regression model analysis of variance (Table 2), which proves that the test results are highly representative. According to the BBD results, the optimal conditions of the extraction process are as follows: enzymatic hydrolysis temperature of 52.79 °C, enzymatic hydrolysis time of 2.01 h, enzymatic hydrolysis pH of 4.93, and solid–liquid ratio of 1:19.57. A verification experiment was carried out under these conditions with slight adjustments: 50 °C enzymatic hydrolysis temperature, 2.0 h reaction time, pH 5, and a 1:20 solid–liquid ratio. Under these modified conditions, the actual total saponin yield was 63.68 ± 0.15 mg/g, which was highly consistent with the yield predicted by the regression model (63.88 mg/g). The results verified that the model is both accurate and adequate for the prediction of the extraction process [38].

### 3.3. Component Analysis

**Figure 4:**
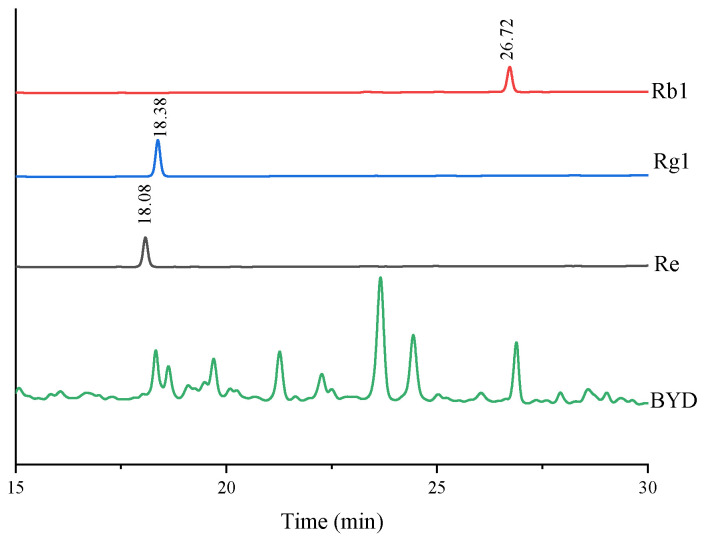
HPLC chromatograms of ginsenoside reference standards and enzymatically hydrolyzed Baoyuan decoction extract (BYD).

In accordance with the Chinese Pharmacopoeia, HPLC was employed for the quantitative analysis of core ginsenosides in the enzymatically hydrolyzed Baoyuan decoction extract. As shown in Figure 4, the results showed that the total content of ginsenoside Re + Rg1 was 4.78 ± 0.49 mg/g (on a dry weight basis), and the content of ginsenoside Rb1 was 4.19 ± 0.27 mg/g. The elution times of ginsenoside Re and Rg1 overlapped, and the chromatographic peaks were partially merged under the established HPLC conditions, which made effective separation difficult; therefore, the two ginsenosides were quantified together. The HPLC quantitative method used in this experiment complied with the relevant requirements of the Chinese Pharmacopoeia, which ensured the accuracy and reliability of the above-mentioned ginsenoside content determination results and provided precise basic data for subsequent research.

### 3.4. Characteristics of Microcapsules

#### 3.4.1. Optimization of Microcapsule Preparation

**Figure 5:**
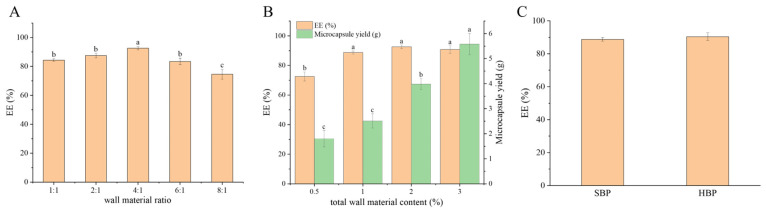
(**A**) Encapsulation efficiency under different wall material ratios (soy protein isolate: pectin); (**B**) encapsulation efficiency and microcapsule yield at different total wall material contents; (**C**) encapsulation efficiency of soy protein isolate–pectin microcapsules (SBP) and high-oleic soy protein isolate–pectin microcapsules (HBP) at 1% total wall material content. Different lowercase letters (a–c) represent significant differences at *p* < 0.05.

As shown in Figure 5, using soy protein isolate (SPI) and pectin as composite wall materials, the effect of the wall material ratio on the encapsulation efficiency of Baoyuan decoction saponins was studied at a fixed total wall material content of 2% (*w*/*v*). With the increasing ratio, the encapsulation efficiency first increased and then decreased, reaching a maximum of 92.56 ± 1.22% at a ratio of 4:1. Excessive SPI led to insufficient pectin for encapsulation, while excessive pectin increased the viscosity and impaired spray-drying encapsulation. Thus, 4:1 was chosen as the optimal ratio. At this ratio, the effects of total wall material content (0.5–3%) were further evaluated. With the increasing total wall material content, the microcapsule yield increased from 1.8 g to 5.6 g, and the encapsulation efficiency reached its maximum at 2%. However, the gummy candies prepared using 1% total wall material content exhibited poor texture. Therefore, a content of 1% was used for the subsequent preparation of gummy candies. To enhance encapsulation without compromising candy texture, high-oleic soy protein isolate (HOSPI) was introduced. At a wall material ratio of 4:1 and a total wall material content of 1%, the encapsulation efficiencies of SBP and HBP were 88.73% and 90.38%, respectively.

#### 3.4.2. Morphological Analysis

**Figure 6:**
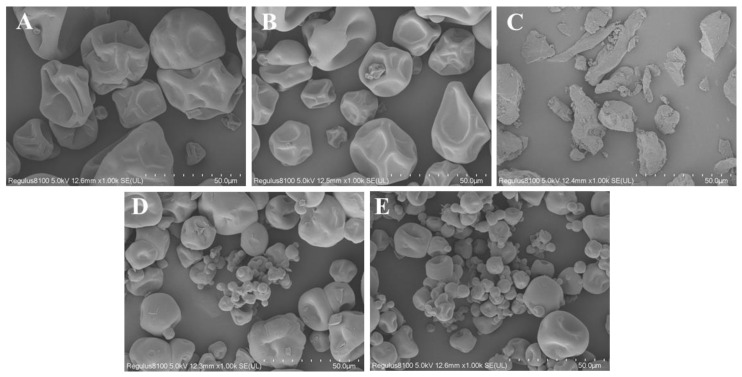
SEM images of different samples. (**A**) Soy protein isolate (SPI); (**B**) high-oleic acid soy protein isolate (HOSPI); (**C**) pectin (PE); (**D**) soy protein isolate–pectin microcapsules (SBP); and (**E**) high-oleic acid soy protein isolate–pectin microcapsules (HBP).

Morphological analysis was conducted on the microcapsules embedded with soy protein isolate (SPI) or high-oleic acid soy protein isolate (HOSPI) and pectin (Figure 6). SPI (Figure 6A) and HOSPI (Figure 6B) exhibited irregular blocky aggregates with densely wrinkled surfaces. Pectin (Figure 6C) presented an amorphous, fragmented structure without distinct particle morphology. Partial wrinkles persisted on the surfaces of both the SBP and HBP composite microcapsules (Figure 6D,E), accompanied by the aggregation of some smaller particles. Compared with SBP microcapsules, HBP microcapsules showed a higher content of small-sized particles and a more uniform particle size distribution. The morphological structure analysis indicated that SPI or HOSPI and pectin might form a more compact composite structure through electrostatic interaction and hydrophobic interaction [39]. The microcapsule structure effectively compensated for the inherent loose arrangement of single-component pectin while further refining the particle morphology of both SPI and HOSPI. Moreover, the compact structure could effectively reduce the exposure of hydrophilic groups, which was consistent with the results of subsequent hygroscopicity and water activity tests (Table 5) [40].

#### 3.4.3. Particle Size, PDI, ζ-Potential, and Encapsulation Efficiency of Microcapsules

**Figure 7:**
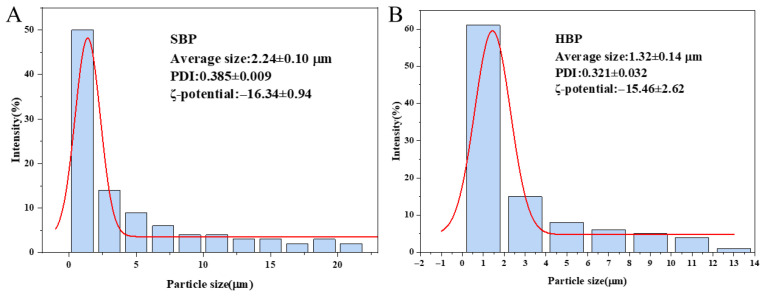
Particle size distribution of SBP (**A**) and HBP (**B**) microcapsules.

The particle size of microcapsules after spray drying are shown in Figure 7. The average particle sizes of soy protein isolate–pectin microcapsules (SBP) and high-oleic acid soy protein isolate–pectin microcapsules (HBP) were 2.24 μm and 1.32 μm, respectively, with corresponding PDIs of 0.385 and 0.321. HBP microcapsules exhibited smaller particle sizes and more uniform particle size distribution, which is consistent with the SEM results. ζ-potential is a crucial parameter for characterizing the stability of microcapsules. The ζ-potentials of SBP and HBP microcapsules were −16.34 mV and −15.46 mV, respectively. Soy protein isolate is electronegative when the environmental pH is above its isoelectric point. Additionally, pectin molecules are electronegative, thus resulting in the overall negative ζ-potential of the composite microcapsules [41,42].

#### 3.4.4. Moisture Content, Water Activity, Solubility, and Hygroscopicity of Microcapsules

As can be seen from Table 5, the moisture content of SBP microcapsules was 3.93 ± 0.15%, while HBP microcapsules was relatively lower (3.58 ± 0.13%). With a water activity (aw) of approximately 0.32 for both microcapsule types, this parameter was lower than the critical thresholds for microbial growth and the physicochemical degradation of microcapsules, which are defined as moisture content < 5% and aw < 0.4 [43,44]. The solubility of HBP and SBP microcapsules was about 97.49% and 91.77%, respectively, indicating that both types of microcapsules possessed favorable solubility. The hygroscopicity of SBP and HBP microcapsules were 6.56 g/100 g and 5.43 g/100 g, respectively. The microcapsules prepared from SPI or HOSPI and pectin exhibit low hygroscopicity, suggesting an enhanced resistance to moisture-induced quality degradation in humid environments [45].

#### 3.4.5. FT-IR Analysis of Microcapsules

**Figure 8:**
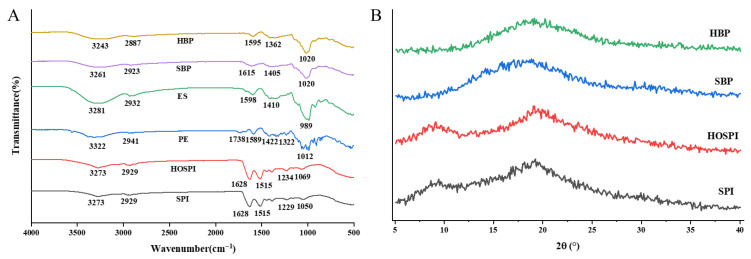
FTIR (**A**) and XRD (**B**) patterns of different samples. SPI: soy protein isolate; HOSPI: high-oleic acid soy protein isolate; PE: pectin; ES: unencapsulated enzymatic hydrolysis extracts; SBP: soy protein isolate–pectin microcapsules; HBP: high-oleic acid soy protein isolate–pectin microcapsules.

The FT-IR results are shown in Figure 8A. In the spectra of SPI and HOSPI, the broad absorption peak at 3273 cm^−1^ indicated the presence of O-H stretching vibrations in SPI and HOSPI. The peak observed at 2929 cm^−1^ was the C-H stretching vibration. The characteristic peaks at 1628 cm^−1^ (amide I band), 1515 cm^−1^ (amide II band), and 1229 cm^−1^ (amide III band) were assigned to the stretching vibrations of the C=O, N-H, and C-H groups, respectively [46]. In the pectin spectrum, the characteristic peaks at 3322 cm^−1^ and 2941 cm^−1^ were typical absorption bands of O-H and C-H groups in polysaccharides. The absorption peak at 1738 cm^−1^ was attributed to the methyl ester group (COOCH_3_). The absorption peak at 1589 cm^−1^ was ascribed to -COO-, which was the characteristic peak of dissociated carboxyl groups in pectin [47]. An absorption band in the range of 1300 to 1450 cm^−1^ was linked to the asymmetric stretching vibration of methyl groups, and the peak at 1012 cm^−1^ was identified as the characteristic signal of the C-O-C stretching vibration in the polysaccharide backbone [28].

The FTIR spectra revealed the interactions between wall materials as well as the molecular interactions between the wall material matrix and the core material. The SBP and HBP microcapsules exhibited absorption peaks at 3261 cm^−1^ and 3243 cm^−1^, respectively, which showed a red shift compared with those of SPI (3273 cm^−1^), HOSPI (3273 cm^−1^), and PE (3322 cm^−1^). This spectral change indicated an increase in hydrogen bonds after microcapsule formation [48]. Compared to the wall materials (SPI or HOSPI), the significant red shift observed in the amide I and amide II bands of both SBP and HBP microcapsules could indicate the potential formation of enhanced electrostatic interactions and hydrogen bonds within the composites, and such strong intermolecular interactions also suggested that the core material was tightly encapsulated in the structure formed by the composite wall materials [18]. The intensity of the C-O-C stretching peak (polysaccharide glycosidic bond) at 1020 cm^−1^ in the spectra of HBP and SBP was lower than that of unencapsulated enzymatic hydrolysis extracts (ESs), which was due to the shielding effect of the composite wall material matrix on the core material functional groups caused by the embedding of ES into the wall material network. It could be inferred that the core material (ES) was successfully embedded in the wall material network, and the stable non-covalent interactions (hydrogen bonds, electrostatic interactions, and hydrophobic interactions) between the wall and core materials laid a structural foundation for the good encapsulation effect and controlled release performance of the microcapsules.

#### 3.4.6. XRD Analysis

The XRD patterns of the raw materials (SPI, HOSPI) and microcapsules are presented in Figure 8B. The crystallinity of microcapsules is closely related to their stability. SPI and HOSPI exhibited two distinct diffraction peaks at approximately 2θ = 9° and 2θ = 20°, which were attributed to the typical crystallization peaks of proteins [49]. The crystallinity of SPI was 40.6%, while that of HOSPI was 38.8%. Both SBP and HBP microcapsules showed a prominent broad peak at 2θ = 20°, while the diffraction peak at 9° disappeared, and their crystallinity decreased significantly to 34.7% and 30.3%, respectively, which suggested that the interaction between wall materials and the core material of saponin disrupted the original crystalline structure of the proteins. It indicated that the formation of protein–pectin microcapsules altered the original molecular arrangement of SPI or HOSPI and reduced the crystallinity of the materials [50]. As revealed by the XRD patterns, the prepared microcapsules exhibited an amorphous structure. Notably, the bioavailability of many amorphous bioactive substances is much higher than that of their crystalline counterparts, and the reduced crystallinity of SBP and HBP microcapsules is expected to improve the release and bioaccessibility of the encapsulated active ingredients [51].

#### 3.4.7. Thermogravimetric Analysis (TGA)

**Figure 9:**
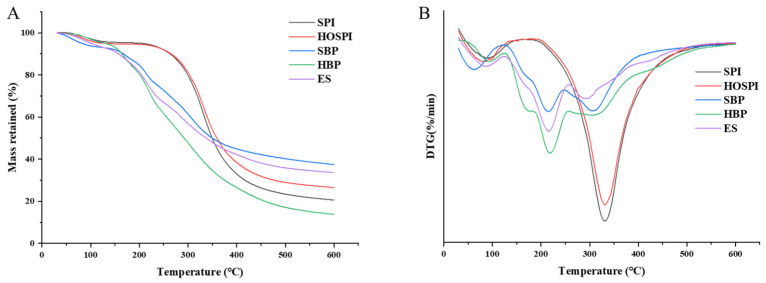
TGA (**A**) and DTG (**B**) curves of different samples. SPI: soy protein isolate; HOSPI: high-oleic acid soy protein isolate; ES: unencapsulated enzymatic hydrolysis extracts; SBP: soy protein isolate–pectin microcapsules; and HBP: high-oleic acid soy protein isolate–pectin microcapsules.

Thermal stability analysis was performed on the raw materials (SPI, HOSPI, and ES) and microcapsules (SBP, HBP) within the temperature range of 30–600 °C. Their thermogravimetric (TG) and derivative thermogravimetric (DTG) curves are shown in Figure 9. All powders exhibited weight loss in the range of 30–150 °C, which corresponded to the evaporation of moisture in the powders. The TG curves of the wall materials (SPI, HOSPI) showed rapid mass loss in the range of 200–400 °C, corresponding to the thermal cracking of the protein backbone (peptide bonds). The DTG curves presented a single peak, indicating that the degradation process was concentrated and no obvious component interaction occurred [28].

As can be seen from the DTG curves, the temperature of the maximum degradation rate (T_max_) of SPI and HOSPI was 330 °C, whereas that of unencapsulated enzymatic hydrolysis extracts and microcapsules reached the maximum at approximately 215 °C. The T_max_ of microcapsules (SBP or HBP) was lower than single wall materials (SPI or HOSPI), which was mainly attributed to two aspects. First, the inherent thermal stability of the unencapsulated enzymatic hydrolysis extract (ES) was lower than that of the wall materials; its thermal degradation at relatively low temperatures would destroy the network of the wall materials and accelerate the overall degradation process. Second, the composite network formed by the wall materials and core materials through hydrogen bonding and hydrophobic and electrostatic interaction altered the conformation of wall material molecules, reduced the activation energy needed for wall material thermal cracking, and shifted the peak degradation rate of the wall materials toward lower temperatures [18,52].

#### 3.4.8. Storage Stability Analysis

**Figure 10:**
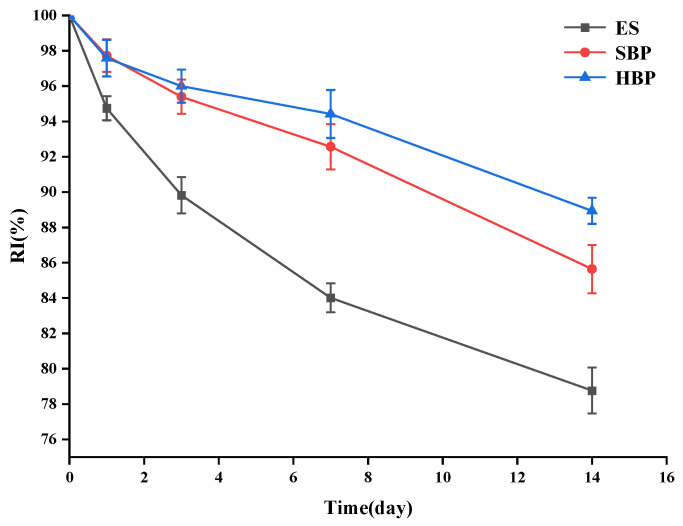
Storage stability of samples at 50 °C. ES: unencapsulated enzymatic hydrolysis extracts; SBP: soy protein isolate–pectin microcapsules; and HBP: high-oleic acid soy protein isolate–pectin microcapsules.

Changes in the total saponin retention rate of SBP and HBP microcapsules under the accelerated storage condition at 50 °C were shown in Figure 10. With the extension of storage time, the unencapsulated saponins degraded rapidly (with a retention rate of 78.8% on the 14th day), whereas the two microcapsules exhibited a relatively slow degradation rate (the retention rates of SBP and HBP were 85.6% and 88.9% on the 14th day), indicating that microcapsules can effectively maintain the structural stability of saponins during storage. During the storage period, the saponin retention rate of HBP was consistently higher than SBP. This difference may be attributed to the stronger binding force between high-oleic acid soy protein isolate and pectin, which forms a denser microcapsule network and thus effectively reduces the thermal degradation of saponin, consistent with the red shifts in amide I and amide II bands in the FTIR results [53].

#### 3.4.9. In Vitro Digestion of Microcapsules

**Figure 11:**
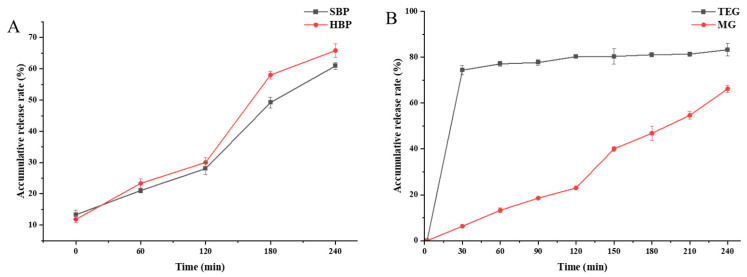
In vitro digestion of microcapsules (**A**) and gummies (**B**). SBP: soy protein isolate–pectin microcapsules; HBP: high-oleic acid soy protein isolate–pectin microcapsules; TEG: unencapsulated gummies; and MG: microcapsule gummies.

The release characteristics of microcapsules in the gastrointestinal tract are crucial for their practical applications. The physical structure of microcapsules affects their physicochemical properties, such as dissolution rate, apparent solubility, and release profile [54]. The digestion and release of saponins from microcapsules were evaluated using the in vitro digestion model. As shown in Figure 11A, the release rate of microcapsules was only approximately 10% during the oral digestion phase, which is inconsistent with the results reported by Zhou et al. [55]. In the simulated gastric digestion phase, the release rates of SBP and HBP reached 21.05% and 23.34%, respectively. The saponin release in the oral phase might be attributed to the dissolution of saponins adhering to the microcapsule surface. During gastric digestion, owing to the steric hindrance effect of pectin, pepsin could only hydrolyze the fraction of proteins uncoated by pectin [56]. Meanwhile, the acidic environment induces hydrophobic aggregation and the hydrogen bond reconstruction of pectin molecules, resulting in a more compact structure. In addition, the pectin coating on the outer layer was resistant to gastric digestive enzymes, which prevented the complete degradation of the protein–pectin shell, thereby retarding the saponin release from microcapsules [18]. In the intestinal digestion phase, the release rates of SBP and HBP increased to 60.92% and 65.83%, respectively, which were significantly higher than that in the gastric phase. In the intestinal environment, a slightly alkaline environment, the pectin molecular chains undergo mild disentanglement, and there was no electrostatic attraction between SPI or HOSPI and pectin molecules, which rendered proteins more susceptible to degradation by intestinal enzymes. This process caused the disruption of the protein–pectin shell and the massive release of active substances [57]. Therefore, the use of soy protein isolates and pectin as composite wall materials for microcapsules can effectively achieve the sustained release of active substances. Meanwhile, compared to SPI, HOSPI as the wall material confers a higher bioaccessibility.

### 3.5. Characteristics of Gummy Candies

#### 3.5.1. Gummy Texture Analysis

Given that gummy candy serves as a highly convenient delivery vehicle for supplementing nutrients and active ingredients, the obtained microcapsules containing plant extracts have been incorporated into gelatin to produce functional gummy candies. The texture profile analysis is shown in Table 6. Compared with the blank gummies and unencapsulated gummies, the microcapsule gummies exhibited higher hardness, gumminess, and chewiness, along with lower adhesiveness, while no significant differences were observed in springiness, cohesiveness, and resilience. These changes in texture properties might be associated with the addition of microcapsules. Specifically, the microcapsule powder replaced part of the water, leading to a reduction in moisture content, which consequently increased the hardness of the candies and endowed them with enhanced chewiness.

#### 3.5.2. In Vitro Digestion of Gummies

Saponins are mainly absorbed in the small intestine. The fraction that is not absorbed in the small intestine will reach the colon, where it is metabolized and absorbed by gut microbiota to exert its biological activity. Hence, protecting saponins and facilitating their targeted release in the intestinal tract is of great significance [58]. Accumulative saponin release rates of unencapsulated gummy candies (TEGs) and microcapsule gummy candies (MGs) during simulated in vitro digestion are shown in Figure 11B. TEG exhibited a burst release in the initial digestion stage (0–30 min), with an accumulative release rate reaching 74.4% at 30 min (gastric digestion) and 83.3% at 240 min (small intestinal digestion). This meant that the unencapsulated active components in gummy candies rapidly dissolved and released in the digestive fluid. In contrast, MG showed a distinct sustained-release profile. The saponin release rate was slow and stable during the gastric digestion phase, with a cumulative release rate of only 23.0% at 120 min; however, the release rate accelerated during the intestinal digestion phase, reaching 66.2% at 240 min. This sustained-release effect is ascribed to the protective effect of the microcapsule wall materials. Furthermore, compared with gummy candies containing unencapsulated extracts, the incorporation of microencapsulated extracts significantly enhances the sustained release of saponins, thereby potentially improving their bioavailability. Although gelatin-based confectionery matrices possess a gelled structure, they do not inherently provide effective controlled release of bioactive compounds [59].

## 4. Conclusions

In this study, an enzymatic extraction method using a combination of cellulase and pectinase was employed to isolate bioactive compounds from Baoyuan decoction. The obtained extract was subsequently microencapsulated via spray drying using a composite wall system of SPI or HOSPI and pectin, which yielded successfully prepared microcapsules. Finally, their application in gummy candies and dynamic in vitro digestion characteristics were evaluated. The results showed that, after RSM optimization, the extraction efficiency of the active components was twice as high as that of water extraction, with a total saponin yield of 63.68 ± 0.15 mg/g. Microcapsules with SPI-PE or HOSPI-PE as wall materials both had high encapsulation efficiencies, which were 88.73% and 90.38%, respectively. Microcapsules can effectively maintain the structural stability of saponins during storage, and they demonstrated a sustained-release effect during in vitro digestion. After being applied to gummy candy, unencapsulated gummies released saponins rapidly (80.2% released during gastric digestion), while microcapsule gummies maintained a favorable sustained-release effect (23.0% released in the stomach and 66.2% in the small intestine). In future work, we will further investigate the sensory properties and storage stability of microencapsulated gummy candies. These findings underscore the considerable potential of microencapsulation technology in advancing functional gummy candy formulations.

## Figures and Tables

**Table 1:** Single-factor experimental conditions for enzyme-assisted extraction.

Single Factor	Tested Conditions
Pectinase concentration (*w*/*w*)	1.0%, 1.5%, 2.0%, 2.5%, 3.0%
Cellulase concentration (*w*/*w*)	1.0%, 1.5%, 2.0%, 2.5%, 3.0%
Solid–liquid ratio (g/mL)	1:10, 1:15, 1:20, 1:25, 1:30
Time (h)	1.0, 1.5, 2.0, 2.5, 3.0
Temperature (°C)	30, 40, 50, 60, 70
pH	3, 4, 5, 6, 7

**Table 2:** Independent variables and levels in Box–Behnken Design (BBD).

Independent Variables	Symbol	Levels		
		−1	0	1
Temperature (°C)	A	40	50	60
Time (h)	B	1.5	2	2.5
pH	C	4	5	6
Solid–liquid ratio (g/mL)	D	1:15	1:20	1:25

**Table 3:** Gradient elution conditions.

Time (min)	Acetonitrile A (%)	Water B (%)
0~35	19	81
35~55	19 → 29	81 → 71
55~70	29	71
70~100	29 → 40	71 → 60

**Table 4:** Analysis of the BBD prediction model.

Source	Sum of Squares	df	Mean Square	F-Value	*p*-Value
Model	163.76	14	11.70	30.99	<0.0001
A-Temperature	17.76	1	17.76	47.06	<0.0001
B-Time	0.2296	1	0.2296	0.6084	0.4505
C-pH	5.14	1	5.14	13.60	0.0031
D-Solid–liquid ratio	4.40	1	4.40	11.67	0.0051
AB	0.5625	1	0.5625	1.49	0.2456
AC	0.3306	1	0.3306	0.8759	0.3678
AD	0.0042	1	0.0042	0.0112	0.9175
BC	2.61	1	2.61	6.91	0.0220
BD	0.0006	1	0.0006	0.0017	0.9682
CD	2.27	1	2.27	6.00	0.0306
A^2^	32.86	1	32.86	87.05	<0.0001
B^2^	36.25	1	36.25	96.04	<0.0001
C^2^	60.15	1	60.15	159.36	<0.0001
D^2^	105.53	1	105.53	279.60	<0.0001
Residual	4.53	12	0.3775		
Lack of Fit	4.46	10	0.4459	12.66	0.0754
Pure Error	0.0705	2	0.0352		
Cor Total	168.29	26			
Std. Dev.	0.6144	R^2^	0.9731		
Mean	57.95	Adjusted R^2^	0.9417		
C.V. %	1.06	Predicted R^2^	0.8464		
		Adeq Precision	19.0434		

**Table 5:** Moisture content, water activity, solubility, and hygroscopicity of SBP and HBP.

	Moisture Content (%)	Water Activity	Solubility (%)	Hygroscopicity (g/100 g)
SBP	3.93 ± 0.15 ^a^	0.323 ± 0.006 ^a^	91.77 ± 0.32 ^a^	6.56 ± 0.13 ^a^
HBP	3.58 ± 0.13 ^a^	0.315 ± 0.003 ^a^	97.49 ± 0.47 ^a^	5.43 ± 0.54 ^b^

Different lowercase letters (a, b) represent significant differences at *p* < 0.05.

**Table 6:** Textural characteristics of gummy candies.

	Hardness (g)	Adhesiveness (g·s)	Springiness	Cohesiveness	Gumminess (g)	Chewiness (g)	Resilience
blank	1137.886 ± 93.347 ^b^	−34.612 ± 2.215 ^b^	0.949 ± 0.010 ^b^	0.872 ± 0.087 ^b^	1024.059 ± 109.529 ^b^	941.629 ± 79.509 ^c^	0.645 ± 0.061 ^a^
TEG	1115.214 ± 118.571 ^b^	−21.286 ± 5.293 ^a^	0.980 ± 0.076 ^a^	0.929 ± 0.020 ^a^	1090.077 ± 43.884 ^ab^	1083.910 ± 29.877 ^b^	0.681 ± 0.026 ^a^
MG	1379.150 ± 63.610 ^a^	−16.331 ± 3.330 ^a^	0.977 ± 0.111 ^a^	0.907 ± 0.031 ^a^	1261.324 ± 73.396 ^a^	1318.116 ± 83.064 ^a^	0.677 ± 0.120 ^a^

Blank: gummies without bioactive components of Baoyuan decoction; TEG: unencapsulated gummies; and MG: microcapsule gummies. (a–c) represent significant differences at *p* < 0.05.

## Data Availability

The original contributions presented in this study are included in the article. Further inquiries can be directed to the corresponding author.
